# Endovascular Treatment of Intracranial Aneurysms: Initial Experience in a Low-Volume Center

**DOI:** 10.5334/jbsr.1918

**Published:** 2020-05-06

**Authors:** Maxime Gudelj, Pierre-Julien Bruyère, Malek Tebache, Laurent Collignon, Boris Lubicz

**Affiliations:** 1 CHR de la Citadelle, Liège, BE; 2 Hopital Erasme, BE

**Keywords:** Intracranial aneurysms, endovascular therapy, education, follow-up

## Abstract

**Objective:**

Endovascular treatment (EVT) is the first-line treatment for ruptured and unruptured intracranial aneurysms (IA). EVT may be performed by interventional neuroradiologist (INR) with different levels of experience. This study aimed at evaluating clinical and anatomic results of IA embolisations performed by a INR with a short experience.

**Materials and Methods:**

Within a 26-month period, 35 IA embolisations were managed by a young INR, 26 of these IA being ruptured. Different EVT techniques were used: coiling alone, stent-assisted coiling and remodeling techniques. Initial angiographic results, clinical outcomes and mid-term anatomic results were evaluated.

**Results:**

Out of 35 procedures, there were seven per-procedural complications leading to one ischemic stroke and one death. Immediate post-procedural complete occlusion was obtained in 91% of procedures (32/35). Good clinical results (modified Rankin Scale Score of 0 or 1) were obtained in 79% of patients (26/33). In a mean follow-up time of 9.5 months, stable occlusion was shown in 88% of IA (21/24).

**Conclusion:**

This study suggests that IA embolisation may be performed by a recently trained INR with good clinical and anatomical outcomes.

## Introduction

The prevalence of intracranial aneurysms (IA) in the general population is approximately 2–6% with an estimated annual rupture risk of 0.7% [1]. Endovascular treatment (EVT) has been proved to be the first-line treatment for ruptured and unruptured IA because it is associated with a lower morbidity and mortality compared to the neurosurgical clipping. Interventional neuroradiologists (INR) have to follow an elite training in order to obtain good outcomes. Nevertheless, this training is poorly defined in the literature. INRs may work in low-volume or high-volume centers, respectively defined as less or equal or more than 50 cases/year [2]. Few data are available over results of IA embolisations performed by recently trained INR who work in a low-volume center. The aim of our study is to report the early experience of a recently trained INR (<3-year experience) working in a low-volume center. We evaluate the initial angiographic results, clinical outcome and mid-term anatomic results.

## Materials and Methods

### Population

Between October 2016 and December 2018, we retrospectively identified in our database all the patients treated for an IA in the radiology department. All clinical and imaging data were prospectively collected. Conventional digital subtraction angiography (DSA) was performed in all our patients. Besides, 3D rotational angiography was obtained in each case in order to fully understand the morphology and size of the aneurysm and the parent artery. Aneurysms were classified according to their morphology (saccular or fusiform) and their size. They were considered as small (<10 mm), large (10–25 mm) or giant (>25 mm). We also classified the neck as wide if the neck/sac ratio was ≥0.7 or if the neck was >4 mm.

Seventy-eight patients were identified with 89 IA. Ninety-five EVT procedures were performed because of 6 of the 89 treated IA reopened over time and were re-treated. Out of the 95 procedures, 60 were performed by a senior INR with a 15-year experience (B.L.) while 35 were managed by a young neuroradiologist with a three-year experience (P.B.). These two patient groups are not comparable as the senior neuroradiologist handled a majority of unruptured IA with most of them having a complex configuration. The young INR treated a greater number of ruptured IA with a more simple morphology. During his training period, the young INR has worked 15 months as second-hand operator for a total of 60 cases and 12 months as a first-hand operator for 115 cases.

### Procedures

All 35 procedures were performed on a monoplane C-arm system under general anesthesia and systemic heparinization. Repeated controls of the activated clotting time (ACT) were carried out in order to assess the efficiency of systemic anticoagulation. In our protocol, we use a 5000 IU bolus infusion of heparin followed by a continuous drip of 1500 IU/h with the aim to double the ACT. At the end of the EVT, heparinization was prolonged for 12–24 hours after the procedure. The patient was then transferred to the intensive care unit where his blood pressure, neurological status and fluid balance were monitored for 24 hours.

The basics of coiling technique for IA occlusion were described by Gugliemi et al. [3]. In most cases, a 6-Fr guiding catheter was used (Envoy, Codman, Miami Lakes, FL). In some patients whose cervical vessels were tortuous, a long 6-Fr introducer was preferred (IVA, Balt, Montmorency, France; or Neuron Max, Penumbra, Alameda, CA; or Cello, Medtronic, Irvine, CA) and was used with an intermediate catheter (Sofia, Microvention, Tustin, CA; or Neuron, Penumbra, Alameda, CA). A microcatheter (XT17, Stryker Neurovascular, Fremont, CA; or Prowler Select, Codman, Miami Lakes, FL; or Headway Duo or 17, Microvention, Tustin, CA) was then used to deliver coils. If needed, the remodeling technique (Hyperglide Balloon, ev3, Irvine, CA) was used [4] or a stent (Lvis Jr, Microvention, Tustin, CA or Leo Stent, Balt, Montmorency, France) was employed to secure the aneurysm neck. Every time a stent was required, for both ruptured or unruptured IA, Clopidogrel was administered for one to three months (75 mg/day). Moreover, patients had to take Aspirin (160 mg/day) for at least six months.

### Clinical Outcomes

Per-procedural and immediate post-procedural complications were recorded. A neurologist examined every patient before and after the procedure. Patients were evaluated again one month after EVT during a specialized neurology consultation. The modified Rankin Scale (mRS) score was used for each patient [5,6].

### Anatomical Outcomes

Immediate post-procedural angiographic acquisitions were evaluated for the quality of aneurysm occlusion. The follow-up imaging protocol includes a conventional DSA at six months, a DSA associated with a magnetic resonance imaging angiography (MRA) at 12 months and then a MRA every year for three years in case of concordance between both techniques. Anatomical results were described by using the Raymond’s classification [7]. The outcome was noted as complete occlusion (class 1 – if no contrast filling the aneurysm), neck remnant (class 2 – persistence of any aneurysmal residue but without opacification of the aneurysmal sac) or incomplete occlusion (class 3 – any residual opacification of the aneurysmal sac). Follow-up studies were assigned to three senior neuroradiologists. On these follow-up imaging examinations, anatomical results were classified as stable (no change of category), minor recurrence (changes from class 1 to class 2) or major recurrence (changes from class 1 or 2 to class 3).

## Results

### Population and Procedures

The mean age of these patients was 53 (range 27–71 years) and 20 patients out of 33 (60%) were women. One patient was treated for three different aneurysms, one ruptured and two unruptured. Among 35 IA, 24 were located in the anterior circulation while 11 were located in the posterior circulation. All IA had a small neck, 24 were small and 11 were large. The mean size of these IA was 7.9 mm (range 3.5–15 mm). Demographic informations and lesion characteristics are summarized in Table 1. Among the 35 procedures performed by the young INR, there were 26 ruptured aneurysms (74%), six unruptured aneurysms (17%) and three recanalizations from a previously coiled aneurysm (9%).

**Table 1:** Population and IAs characteristics.

	Ruptured IA (%)	Unruptured IA (%)	Recanalizations (%)

**No. patients**	26	5	3
**Male/Female**	13/13	0/5	0/3
**Mean Age (years)**	53,3	52	60
**mRS Score**			
0–2	19	4	3
3–5	3	2	0
6 (death)	4	0	0
**No. IA**	26 (74)	6 (17)	3 (9)
**Size**			
Small (<10 mm)	18 (51)	5 (14)	1 (3)
Large (10–25 mm)	8 (23)	1 (3)	2 (6)
Giant (>25 mm)	0	0	0
**Localization**			
MCA	3 (9)	1 (3)	0
AcomA	14 (40)	2 (5.5)	0
PcomA	5 (14)	2 (5.5)	0
BT	1 (3)	0	2 (5.5)
PICA	1 (3)	0	0
PeriA	0	1 (3)	0
ICA	2 (5.5)	0	1 (3)

Abbreviations: AcomA, anterior communicating artery; PcomA, posterior communicating artery; BT, basilar trunk; MCA, middle cerebal artery; PICA, posterior inferior cerebellar artery; ICA, Internal carotid artery.

All aneurysm embolizations were performed with coils. Out of 35 procedures, five required the use of a balloon catheter. In four of them, the aim was to obtain a denser coils packing. In one case, the balloon was employed because of a per-procedural aneurysm rupture. Three of these aneurysms were located on the posterior communicating artery, one was located on the carotid-ophthalmic artery and one was found on the M1 segment of the middle cerebral artery. Stent assisted coiling (SAC) technique was executed in six cases (5 Lvis Jr and 1 Leo stent). In four of these procedures, SAC was carried out as the aneurysm morphology and/or neck/sac ratio were considered unfavorable. In the remaining two cases, coils protruded in the parent arteries and resulted in thrombus formation. Deployment of a stent and administration of intravenous (IV) abciximab were used to deal with those issues. This was successful in one case while in the other, the treatment failed and led to infarction in the left anterior cerebral artery territory. Regarding the complications of EVT with coiling alone, we noted three thrombus formation in the anterior circulation and one carotid dissection. Thrombi fully disappeared after IV abciximab injection. The dissection was 1-mm-thick and 10-mm-long, so conservative management was preferred and it faded out on further imaging studies without any clinical repercussion. Overall, there were seven per-procedural complications (20%) leading to one ischemic stroke and one death (see Table 2).

**Table 2:** Procedural and periprocedural complications.

Patient age	IA status	IA size (mm)	IA localization	Complication	Complication treatment	Treatment efficiency	Clinical results (mRS)

**31**	R	10	AcomA	Carotid dissection	Conservative	Complete resolution	0
**69**	R	10	PcomA	Intraprocedural rupture	Balloon assisted coiling	Inefficient	6 (death)
**56**	R	8.5	BT	Gail protrusion & thrombus in the parent artery	Stent & abciximab	Complete resolution	0
**43**	R	7	PcomA	Thrombus	Abciximab	Complete resolution	1
**50**	R	6.5	AcomA	Thrombus	Abciximab	Complete resolution	0
**45**	R	7	AcomA	Thrombus	Abciximab	Complete resolution	0
**62**	R	8	AcomA	Coil protrusion & thrombus in the parent artery	Stent & abciximab	Inefficient	5 (left frontal infarction)

Abbreviations: AcomA, anterior communicating artery; PcomA, posterior communicating artery; BT, basilar trunk; R, ruptured aneurysm.

### Clinical Outcomes

Among 33 patients, 24 (73%) presented excellent clinical results (mRS = 0). Two patients (6%) admitted for ruptured aneurysms kept a slight upper limb paresis but remain independent (mRS = 1). One of these two patients already had symptoms on admission. Three patients (9%) were dependent with mRS scores of 4 or 5. In two of them, this score was already present before the procedure. One patient conserved a moderate paretic dysarthria, a severe left hemiplegia with a strong spasticity of the left upper limb and a moderate left hyposensibility. The third patient had a mRS score of 0 on admission but EVT was complicated with thrombus formation resulting in infarction in the left anterior cerebral artery territory. Four patients (12%) died early after EVT. In one of them, aneurysm rupture occurred during the procedure. This patient had a large subarachnoid hemorrhage, grade IV according to the modified Fisher Scale [8], and developed a vasospasm with cerebellous, frontal, parietal and occipital ischemic lesions on MRI studies. Another patient died from multiple organ failure.

### Anatomical Outcomes

Immediate post-procedural DSAs showed 32 complete occlusions (91%) and three neck remnants (9%). The latter three cases were treated with coiling alone. There was no incomplete occlusion in our series. Follow-up imaging studies were obtained in 23 patients with 24 IAs. Mean follow-up time was 9.5 months (range 1–18 months). The follow-up imaging could be unavailable for many reasons such as death (four patients), lost to follow-up (two patients) or recently performed procedures (four patients).

Stable occlusions were showed in 21 cases (88%). Minor recurrence was identified in one case (4%) and major recurrence was depicted in two cases (8%). Of these two latter cases, one was a large recanalized basilar tip aneurysm that had been previously treated with coils. The other case was a small ruptured anterior communicating artery aneurysm. This aneurysm was treated with bare coils and immediate post-procedural DSA showed a neck remnant. There was no rebleeding within the time frame of the follow-up.

## Discussion

This study illustrates the early experience of a recently trained young INR working in a low-volume center. To our knowledge, this is the first paper dealing with that kind of experience in Belgium. There are few studies evaluating learning curves for IA embolizations in the literature. One of these studies demonstrated that the risk of complications significantly decreases with physician experience in the setting of unruptured aneurysms treated with coil embolization [9]. The first-year experience of interventional neurologists was published in another study [10]. It showed successful treatments of both ruptured and unruptured aneurysms with morbidity and mortality rates comparable to those found in other studies. Interventional neuroradiology requires advanced technical skills and a deep knowledge within a large range of vascular pathologies, devices and procedures. Up till now, there was no established training program for this recent subspecialty in Belgium. Since late 1990s, following guidelines support residency/fellowship education in interventional neuroradiology in North America [11].

Aneurysm embolization was performed in all 35 cases assigned to the recently trained INR. Intraprocedural complications occurred in 20% of the cases with procedure-related morbidity and mortality rates of 3%, respectively. It is quite similar to the rate of procedure-related complications found in the literature [10,12]. The initial anatomical results are excellent with 100% of immediate adequate occlusions (class 1 and 2) whereas the rate is around 91–94% in the literature [12]. The aneurysmal recurrence rate is also in accordance with previous studies [7,12]. Regarding the clinical outcome, good results were observed in 26 patients (79%) with a mRS score of 0 or 1 at discharge. One of these patients was admitted with a mRS score of 5. Three patients (9%) were dependent with a mRS score of 4 or 5 at discharge and four patients (12%) were deceased (mRS = 6). These clinical results are comparable to those published in Clarity and ISAT studies [13,14]. They are slightly better than those reported in other studies [12]. This could be explained by the fact that this study does not only include ruptured IA but also unruptured IA and recanalizations of previously treated IA.

This retrospective study bears limitations. First, the group of patients is inhomogeneous as we included a majority of ruptured IA but also some unruptured IA and recanalizations. This could affect the results presented in this study as we know that these categories of IA have different biological behaviors. Indeed, a study demonstrated the lower rigity of the wall in ruptured IA, which can potentially affect recurrence and complication rates [15]. On the other hand, procedures were also heterogenous, including simple coiling, remodeling technique and stent-assisted coiling. Procedure-related complications are influenced by the technique. The population of our study is quite small and length of the imaging follow-up is limited (only nine patients had an imaging exam at 12 months and above). Lastly, our good clinical and anatomical results could partially be explained by the absence of giant aneurysms and wide neck aneurysms.

## Conclusion

This study suggests that a recently trained INR working in a low-volume center can successfully carry out IA embolization with morbidity and mortality rates similar to those described in previous studies. Further investigation is needed to confirm these clinical and anatomical results.
